# Long term trends and spatial distribution of animal bite injuries and deaths due to human rabies infection in Uganda, 2001-2015

**DOI:** 10.1371/journal.pone.0198568

**Published:** 2018-08-21

**Authors:** Ben Masiira, Issa Makumbi, Joseph K. B. Matovu, Alex Riolexus Ario, Immaculate Nabukenya, Christine Kihembo, Frank Kaharuza, Monica Musenero, Anthony Mbonye

**Affiliations:** 1 Department of National Disease Control, Uganda Public Health Fellowship Program, Ministry of Health, Kampala, Uganda; 2 Department of National Disease Control, Ministry of Health, Kampala, Uganda; 3 Makerere University College of Health Sciences, Makerere School of Public Health, Department of Disease Control and Environment, Makerere University, Kampala, Uganda; 4 Makerere University College of Veterinary Medicine, Animal Resources and Biosecurity, Makerere University, Kampala, Uganda; 5 Programs Department, African Field Epidemiology Network, Kampala, Uganda; 6 Department of Community Health, Ministry of Health, Kampala, Uganda; Wistar Institute, UNITED STATES

## Abstract

**Background:**

In the absence of accurate data on trends and the burden of human rabies infection in developing countries, animal bite injuries provide useful information to bridge that gap. Rabies is one of the most deadly infectious diseases, with a case fatality rate approaching 100%. Despite availability of effective prevention and control strategies, rabies still kills 50,000 to 60,000 people worldwide annually, the majority of whom are in the developing world. We describe trends and geographical distribution of animal bite injuries (a proxy of potential exposure to rabies) and deaths due to suspected human rabies in Uganda from 2001 to 2015.

**Methods:**

We used 2001–2015 surveillance data on suspected animal bite injuries, collected from health facilities in Uganda. To describe annual trends, line graphs were used and linear regression tested significance of observed trends at *P*<0.05. We used maps to describe geographical distribution of animal bites by district.

**Results:**

A total of 208,720 cases of animal bite injuries were reported. Of these, 27% were in Central, 22% in Eastern, 27% in Northern and 23% in Western regions. Out of 48,720 animal bites between 2013 and 2015, 59% were suffered by males and 81% were persons aged above 5 years. Between 2001 and 2015, the overall incidence (per 100,000 population) of animal bites was 58 in Uganda, 76 in Northern, 58 in Central, 53 in Western and 50 in Eastern region. From 2001 to 2015, the annual incidence (per 100,000 population) increased from 21 to 47 (*P = 0*.*02*) in Central, 27 to 34 (*P = 0*.*04*) in Eastern, 23 to 70 (*P = 0*.*01*) in Northern and 16 to 46 (*P = 0*.*001*) in Western region. A total of 486 suspected human rabies deaths were reported, of which 29% were reported from Eastern, 28% from Central, 27% from Northern and 17% from Western region.

**Conclusion:**

Animal bite injuries, a potential exposure to rabies infection, and mortality attributed to rabies infection are public health challenges affecting all regions of Uganda. Eliminating rabies requires strengthening of rabies prevention and control strategies at all levels of the health sector. These strategies should utilize the “One Health” approach with strategic focus on strengthening rabies surveillance, controlling rabies in dogs and ensuring availability of post exposure prophylaxis at lower health facilities.

## Introduction

Rabies remains a public health challenge in many parts of the world with over 90% of human rabies cases worldwide attributed to dog bites [1–3]. Rabies is one of the most deadly infectious diseases in the 21^st^ century, with a case fatality rate approaching 100% [1]. However, the burden and trends of human rabies infection in developing countries is poorly understood and underestimated due to lack of accurate data, poor surveillance systems, underreporting and rabies misdiagnosis [4–6]. Animal bites in humans provide an important source of epidemiological information which is crucial in enhancing rabies surveillance in humans and animals and allocation of resources [7]. Despite the availability of effective post exposure vaccine treatment among humans and effective vaccines among animals, rabies is estimated to kill 50,000 to 60,000 people worldwide each year and it was responsible for 1,460,000 Disability Adjusted Life Years in 2010 [2, 8]. The majority of rabies deaths occur in developing countries especially in tropical regions of Africa, South America, Asia and Oceania [9]. Between 1993 and 1998, 15 cases of human rabies infection were reported annually in Uganda [10–16].

Rabies virus causes a clinical syndrome of an acute encephalitis or meningoencephalitis. The virus itself is a single-stranded, negative-sense, RNA virus in the genus Lyssavirus, family Rhabdoviridae. Rabies virus is transmitted to humans through a bite, scratch or mucous membrane exposure to a rabid animal. The virus replicates locally at the site of injury and gains access to the central nervous system [17, 18]. The typical route involves entry via the motor end-plate into motor nerve axons, where the virus can then travel to the spinal cord and brain. There, a productive infection takes hold, and the virus travels outwards to innervated organs including most notably the salivary glands. Thus, bite injuries are the most common first step in the development of human rabies infections.

Domestic dog bites are the most important source of infection to humans [1, 2] and they remain a serious public health challenge throughout the world. A survey conducted between 2001 and 2003 in the United States found that up to 4.5 million dog bites were reported each year [19]. Despite scanty data about the incidence of animal bites in the African region, there is evidence to show that the burden of animal bites is high in this region too. For example, in 1998, up to 413,450 received post exposure treatment in Africa of which 94% were exposed to domestic dogs and in Tanzania 23,709 suspected rabid dog bite injuries were reported between 1990 and 1996 [7, 20]. A health facility passive surveillance study conducted in Uganda estimated that 6,601 dogs bites are inflicted on humans each year [21].

Although human rabies has been largely eliminated in the United States of America, Western Europe, Japan and Malaysia, it remains an under-reported public health problem in the African region [20, 22]. There is limited data about the actual incidence of human rabies infection in most African countries yet this information is essential in planning and implementation of effective control strategies. Most of these countries generally have bottlenecks in their surveillance systems, reporting of epidemiological data and they lack routine laboratory diagnostic services and in many cases patients do not report to health facilities for treatment [7, 14, 23]. In addition to that, many patients opt to seek treatment options outside modern medicine such as traditional healers because of the association of rabies symptoms to witchcraft [24].

Due to the challenges of diagnosis of rabies in developing countries, surveillance of animal bites was initiated in Uganda to provide information about potential exposure to rabies infection and to inform the post-exposure prophylaxis program. The objective of the study was to describe long term trends and geographical distribution of animal bite injuries and suspected rabies deaths in Uganda from 2001 to 2015, using epidemiological surveillance data.

## Methods

### Study site

**Fig 1:**
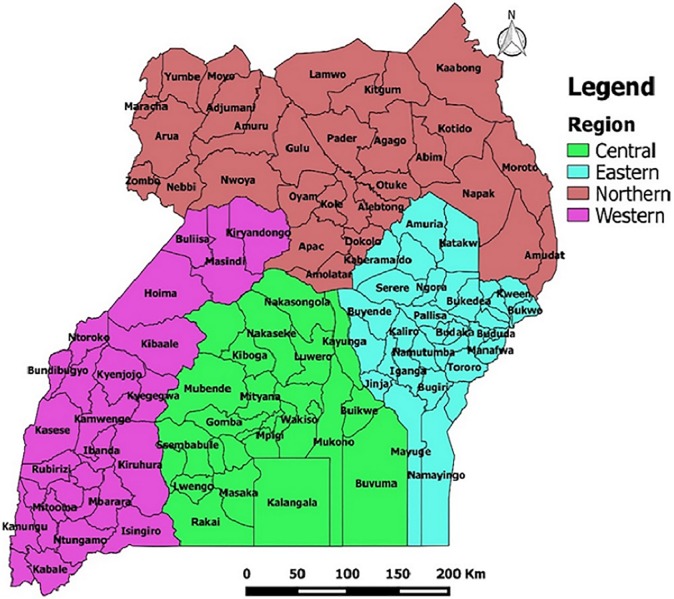
Location of Uganda districts by region.

The study sites were 2,521 health facilities in 112 districts of Uganda that submitted data to the national weekly epidemiological surveillance database, from 2001 to 2015. During the study period, the number of districts in Uganda increased from 56 in 2001 to 112 in 2015. Similarly, the number of health facilities increased from 2,214 in 2001 to 2,521 in 2015, as more districts were created. In this study, the districts were grouped into four regions in order to examine and compare the data. These regions included the Central, Eastern, Northern and Western (Fig 1. Location of Uganda districts by region).

### Study population

The study population included all new patients diagnosed with animal bite injuries and patients who died due to suspected rabies infection at health facilities. This data was extracted from patient registers at the health facilities and submitted to the Ministry of Health (MoH).

### Design

The study was a retrospective review of epidemiological surveillance data on animal bite injuries and suspected rabies deaths reported to the national database from 2001 to 2015.

### Surveillance system for animal bites and rabies in Uganda

Animal bite injuries inflicted on humans were used to track the potential risk of exposure to rabies in Uganda. Reporting of animal bite injuries and suspected rabies deaths from districts to Uganda MoH started in 2001. Animal bite injuries and human deaths due to suspected rabies were diagnosed by clinicians at health facilities, using standard case definitions provided by the MoH. An animal bite was defined as exposure to a bite from an animal that could potentially transmit rabies. A suspected rabies case was defined as a person with history of contact with a rabid animal with one or more of the following: headache, neck pain, nausea, fever, fear of water, anxiety, agitation, abnormal tingling sensations or pain at the wound site. There is no routine laboratory surveillance or diagnostic capacity for human rabies in Uganda.

Each health facility summarized its data on animal bite injuries and suspected rabies deaths every week and every month using Heath Management Information System (HMIS) data collection tools and submitted this information to the district which submitted it to the national level. Data about the actual age and details of the biting animal was not reported to the national level because of lack of these indices in the data collection tools.

Between 2001 and 2011, Uganda relied on paper-based HMIS whereby HMIS reports on priority diseases were made at health facilities and delivered to the office of the District Health Officer (DHO). The DHO’s office summarized all health facility reports into one district weekly/monthly surveillance report which was sent to the MoH where the information was entered into the national database. In early 2011, the District Health Information System Software Version 2 (DHIS-2) was introduced to enable districts to upload their HMIS reports directly [25]. At the end of 2011, a new platform known as mTrac was launched within the DHIS-2 to enable health facilities to use mobile phone messaging to directly submit their weekly epidemiological surveillance reports to the national database after review and confirmation from the DHO’s office [26].

### Data extraction and analysis

We extracted data on the number of animal bite injuries and suspected rabies deaths by period (year), region and district. Data about the gender and age group of victims of animal bites and suspected rabies deaths was extracted for the period between 2013 and 2015 because this data was not collected prior to this period. The age of cases of animal bites and suspected rabies deaths were reported using two broad age groups (i.e. below 5 years and ≥5 years). We extracted population data from the Uganda National Census 2002 and 2014 [27, 28].

We used Excel 2010 to generate line graphs, STATA-12 to conduct statistical analyses and Quantum Geographic Information System (QGIS) to generate maps. Annual populations were estimated using a growth rate of 3.03% [27]. Proportions of animal bite injuries by age (<5years and ≥5 years) and sex were computed for the period of 2013 to 2015. Incidence (per 100,000 population) was calculated by using number of animal bite injuries as a numerator and the total district, region or national population as a denominator. Line graphs were used to demonstrate annual trends of incidence and deaths from 2001 to 2015. We used linear regression to assess trends of incidence of animal bites and deaths due to rabies at a significance level of P<0.05. Geographical differences in the incidence of animal bite injuries across districts were illustrated using colorimetric maps, using five year intervals i.e. 2001–2005; 2006–2010; 2011–2015. Five year intervals were used to cater for the new districts which were introduced between 2001 and 2015. We categorized district specific incidence (cases per 100,000 population) into four main categories, namely, <25, 25–49.9, 50–74.9 and ≥75.

### Ethical considerations

The Ministry of Health of Uganda gave permission to use the data and approved the study design. This study was approved by Makerere University School of Public Health Higher Degrees Research and Ethics Committee and the National Council of Science and Technology.

## Results

### Characteristics of patients with animal bite injuries; 2001–2015

From 2001 to 2015, a total of 208,720 patients with animal bite injuries were treated at health facilities across the country. Of these, 27.4% (57,252) were in Central region, 22.4% (46,742) in Eastern region, 27.0% (56,382) in Northern region and 23.2% (48,344) in Western region (Table 1).

**Table 1:** Cases of animal bite injuries by region; 2001–2015.

Year	Animal bite injuries (row %)				
	Central	Eastern	Northern	Western	Overall
2001	1,330 (27.0)	1,601 (32.5)	1,049 (21.3)	953 (19.3)	4,933 (100)
2002	2,194 (28.8)	2,055 (27.0)	1,964 (25.8)	1,392 (18.3)	7,605 (100)
2003	2,601 (25.6)	2,487 (24.4)	3,126 (30.7)	1,958 (19.2)	10,172 (100)
2004	2,963 (25.3)	2,607 (22.3)	3,188 (27.2)	2,947 (25.2)	11,705 (100)
2005	3,296 (26.1)	2,958 (23.4)	3,092 (24.5)	3,272 25.9)	12,618 (100)
2006	4,381 (32.3)	2,750 (20.3)	3,320 (24.5)	3,105 (22.9)	13,556 (100)
2007	4,341 (28.3)	3,672 (24.0)	3,896 (25.4)	3,411 (22.3)	15,320 (100)
2008	4,725 (28.9)	3,631 (22.2)	4,544 (27.8)	3,434 (21.0)	16,334 (100)
2009	4,175 (24.7)	4,148 (24.6)	4,641 (27.5)	3,923 (23.2)	16,887 (100)
2010	4,633 (26.4)	4,519 (25.8)	4,097 (23.4)	4,269 (24.4)	17,518 (100)
2011	4,684 (29.9)	3,078 (19.7)	4,213 (26.9)	3,683 (23.5)	15,658 (100)
2012	4,008 (24.6)	3,581 (22.0)	4,743 (29.1)	3,954 (24.3)	16,286 (100)
2013	4,230 (28.5)	2,819 (19.0)	4,031 (27.1)	3,778 (25.4)	14,858 (100)
2014	5,030 (27.8)	3,710 (20.6)	5,274 (29.2)	4,059 (22.5)	18,073 (100)
2015	4,661 (27.1)	3,126 (18.2)	5,204 (30.3)	4,206 (24.5)	17,197 (100)
**2001–2015**	**57,252 (27.4)**	**46,742 (22.4)**	**56,382 (27.0)**	**48,344 (23.2)**	**208,720 (100)**

Information on gender and age group was available on 48,720 animal bites reported from 2013 to 2015. When disaggregated by sex, the proportion of animal bites was higher among males than that among females across all regions; 59% vs. 41% in Central region (*P*<0.001), 65% vs. 35% in Eastern region (*P*<0.001), 67% vs. 33% in Northern region (*P*<0.001) and 61% vs. 39% in Western region (*P*<0.001) (Table 2). Up to 81% (n = 39,618) were patients ≥5 years of age and 19% (n = 9,102) were below 5 years of age (Table 2). During this period, the proportion of persons below 5 years with animal bite injuries was highest in the Northern region (24%) followed by the Central region (21%), Eastern region (15%) and Western region (11%). The proportion of animal bite injuries among children below 5 years significantly increased from 11% to 35% in Central region (*P*<0.001), 11% to 28% in Northern region (*P* = 0.003), 6% to 15% in Western region (P<0.001) and decreased from 16% to 6% in Eastern region (*P* = 0.04).

**Table 2:** Characteristics of animal bite injuries by gender, age and region; 2013–2015.

Period	Gender/age group	Region				Overall (%)
		Central (%)	Eastern (%)	Northern (%)	Western (%)	
**2013**	Male	2,974 (70.3)	927 (56.1)	2,665 (70.3)	2,169 (57.4)	8,735 (64.9)
	Female	1,256 (29.7)	725 (43.9)	1,126 (29.7)	1,609 (42.6)	4,716 (35.1)
	<5 years	457 (10.8)	261 (15.8)	409 (10.8)	242 (6.4)	1,369 (10.2)
	≥ 5 years	3,773 (89.2)	1,391 (84.2)	3,382 (89.2)	3,536 (93.6)	12,082 (89.8)
**2014**	Male	2,739 (54.5)	2,708 (73.0)	3,106 (58.9)	2,191 (54.0)	10,744 (59.5)
	Female	2,291 (45.5)	1,002 (27.0)	2,168 (41.1)	1,867 (46.0)	7,328 (40.5)
	<5 years	997 (19.8)	801 (21.6)	1,545 (29.3)	479 (11.8)	3,822 (29.0)
	≥ 5 years	4,033 (80.2)	2,909 (78.4)	3,729 (70.7)	3,579 (88.2)	14,250 (71.0)
**2015**	Male	2,498 (53.6)	1,910 (61.1)	3,793 (72.9)	3,009 (71.5)	11,210 (65.2)
	Female	2,163 (46.4)	1,216 (38.9)	1,411 (27.1)	1,197 (28.5)	5,987 (34.8)
	<5 years	1,610 (34.5)	175 (5.6)	1,478 (28.4)	648 (15.4)	3,911 (22.7)
	≥ 5 years	3,051 (65.5)	2,951 (94.4)	3,726 (71.6)	3,558 (84.6)	13,286 (77.3)
**2015–2016**	Male	8,211 (59.0)	5,545 (65.3)	9,564 (67.0)	7,369 (61.2)	30,689 (58.9)
	Female	5,710 (41.0)	2,943 (34.7)	4,705 (33.0)	4,673 (38.8)	18,031 (41.1)
	<5 years	3,064 (21.2)	1,238 (14.6)	3,432 (24.1)	1,369 (11.4)	9,102 (18.7)
	≥ 5 years	10,857 (78.8)	7,251 (85.4)	10,837 (75.9)	10,673 (88.6)	39,618 (81.3)

### Trends of incidence and reporting of animal bite injuries from 2001 to 2015

**Fig 2:**
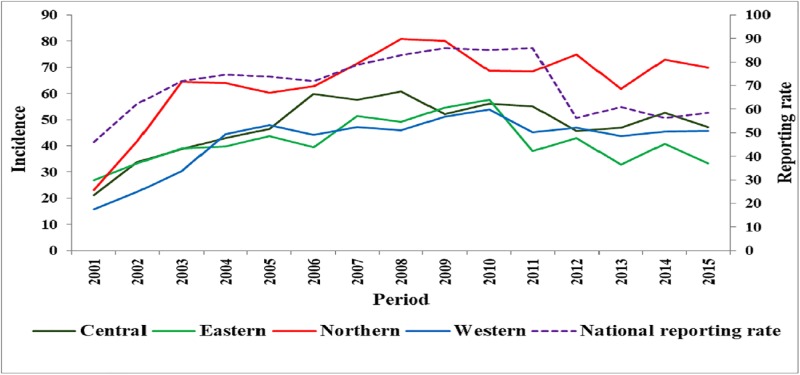
Trends in the incidence of animal bites by region; 2001–2015.

Between 2001 and 2015, the overall incidence (per 100,000 population) of animal bites in Uganda was 58.1 cases per year. The Northern region had the highest overall incidence of 75.7 compared to 58.0 in Central, 52.5 in Western and 49.9 in Eastern region. From 2001 to 2015, the incidence of animal bites significantly increased from 21.2 to 47.2 (*P = 0*.*02*) in Central, 26.8 to 33.8 in Eastern (*P = 0*.*04*), 23.1 to 69.9 in Northern (*P = 0*.*01*) and 15.8 to 45.7 in Western region (*P = 0*.*01*) (Fig 2. Trends in the incidence of animal bites by region; 2001–2015).

The overall reporting rate for weekly epidemiological surveillance reports was 70.4% at the national level (Table 3). However, reporting rates varied across the different regions of the country; Central (68.2%), Eastern (74.2%), Northern (74.1%) and Western (65.1%).

**Table 3:** Weekly epidemiological reporting rates by region; 2001–2015.

Year	Central	Eastern	Northern	Western	Overall
2001	50.9	47.9	45.1	46.2	47.5
2002	60.1	68.3	53.7	45.0	56.8
2003	74.6	73.3	56.4	47.2	62.9
2004	77.2	78.7	66.1	59.1	70.3
2005	75.7	80.5	72.3	61.4	72.5
2006	76.4	84.5	75.4	73.3	77.4
2007	75.4	81.8	82.3	70.9	77.6
2008	74.4	85.4	93.1	73.3	81.5
2009	79.1	89.0	94.5	80.4	85.7
2010	73.2	84.8	92.8	81.0	82.9
2011	80.8	87.6	91.4	85.4	86.3
2012	60.4	80.3	87.5	71.5	74.9
2013	60.5	59.2	64.2	62.0	61.5
2014	53.0	52.2	62.7	57.0	56.2
2015	50.6	59.7	74.5	63.3	62.0
2001–2015	68.2	74.2	74.1	65.1	70.4

### Geographical distribution of animal bite injuries from 2001 to 2015

**Fig 3:**
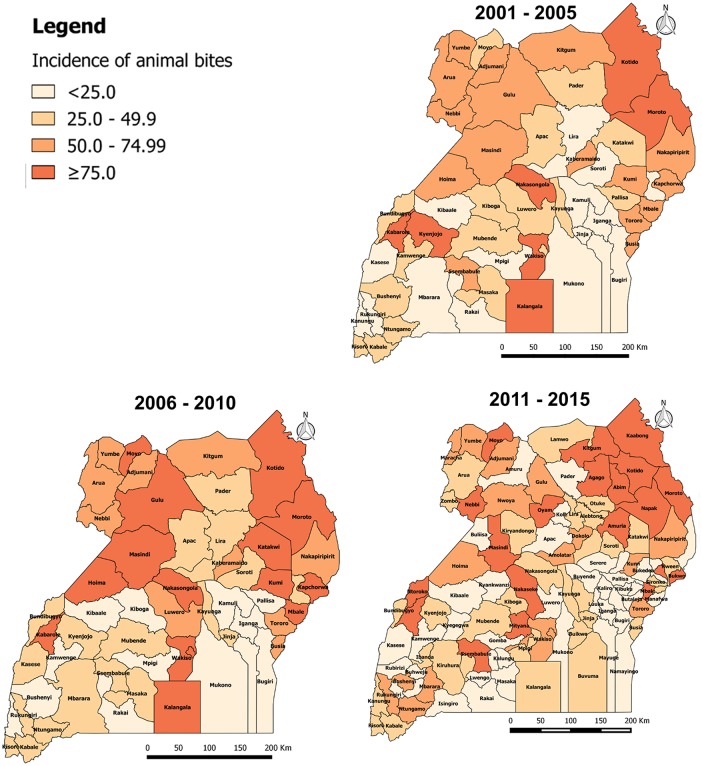
Annual district incidence (per 100,000) of animal bites from 2001 to 2005.

From 2001 to 2015, the annual incidence of animal bites injuries was persistently high in districts of the Northern region of Uganda. From 2001 to 2005, the annual incidence of animal bite injuries in Uganda was 47.8 per 100,000 population. The incidence was ≥75 animal bites per 100,000 population in 7 out of 56 districts (12.5%) i.e. Kotido (217), Wakiso (130), Moroto (122), Kabarole (114), Nakasongola (100), Kyenjojo (76) and Kalangala (76). Sixteen out of 56 districts (29%) had an incidence of 50.0–74.9 animal bites per 100,000, of which 44% were in Northern, 38% in Eastern, 13% in Western and 6% in Central (Fig 3. Annual district incidence (per 100,000) of animal bites from 2001 to 2005).

**Fig 4:**
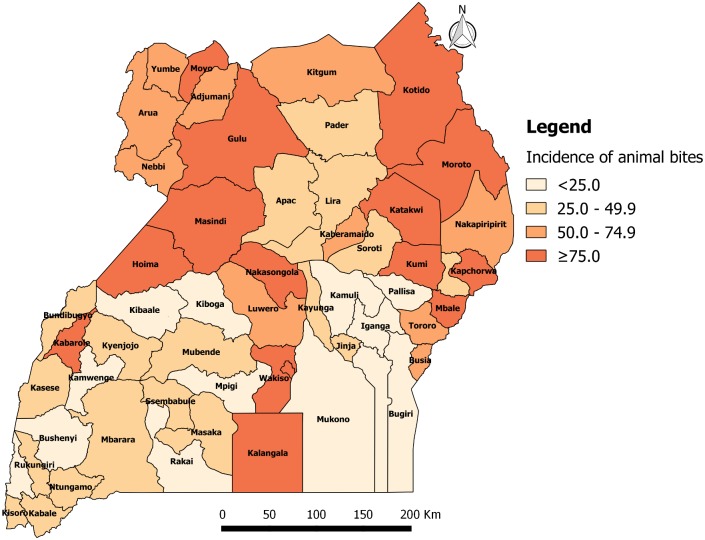
Annual district incidence (per 100,000) of animal bites from 2006 to 2010.

From 2006 to 2010, the annual incidence of animal bites in Uganda was 59.1 per 100,000 population. The incidence was ≥75 animal bites per 100,000 population in 15 out 56 districts (27%) i.e. Kotido (351), Wakiso (173), Moroto (150), Kapchorwa (134), Kabarole (126), Kumi (105), Kalangala (103), Hoima (98), Masindi (92), Kampala (89), Moyo (84), Nakasongola (79), Mbale (79), Gulu (78) and Katakwi (77). Ten out of 56 districts (18%) had an incidence of 50.0–74.9 animal bites per 100,000, of which 60% were in Northern, 30% in Eastern and 10% in Central (Fig 4. Annual district incidence (per 100,000) of animal bites from 2006 to 2010).

**Fig 5:**
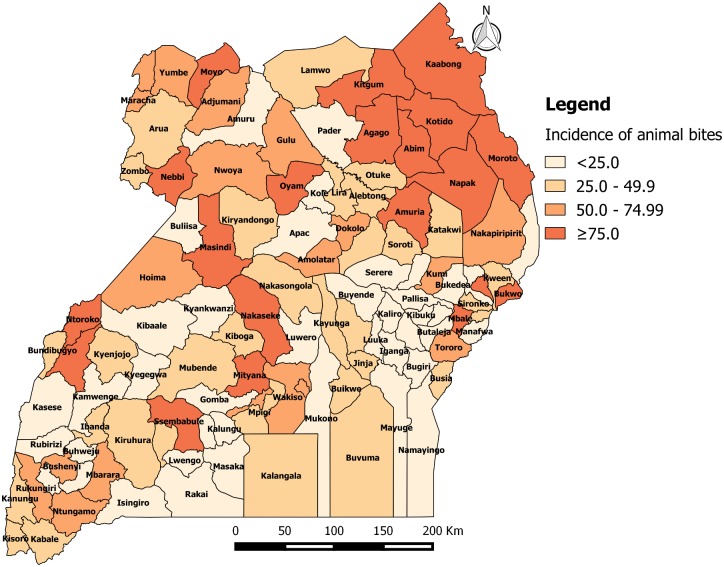
Annual district incidence (per 100,000) of animal bites from 2011 to 2015.

From 2011 to 2015, the overall incidence of animal bites in Uganda was 51.3 per 100,000 population. The incidence was ≥75 animal bites per 100,000 population in 20 out 112 districts (18%). Nineteen out of 112 districts (17%) had an incidence of 50.0–74.9 animal bites per 100,000 of which, 47% were in Northern, 26% in Western, 16% in Central and 11% in Eastern (Fig 5. Annual district incidence (per 100,000) of animal bites from 2011 to 2015).

### Dog population in Uganda

Results of the national livestock census that was conducted in 2008 [29] showed that Uganda had a total of 1,580,930 dogs of which 457,690 (29.0%) was in Northern region, 440,400 (27.9%) in Western region, 370,830 (23.4%) in Central region and 312,010 (19.7%) in Eastern region (Table 4).

**Table 4:** Distribution of domestic dogs by region.

Region	Number of dogs	Percent
Central	370,830	23.9
Eastern	312,010	19.7
Northern	457,690	29.0
Western	440,400	27.9
**OVERALL**	**1,580,930**	**100**

### Deaths due to suspected human rabies infection from 2001 to 2015

Between 2001 and 2015, a total of 486 suspected human rabies deaths were diagnosed at health facilities in the country, representing an average of 32 deaths annually. Out of these deaths, 28.6% were reported from the Eastern region, 27.8% from Central region, 26.7% from Northern region and 16.9% from Western region (Table 5). Out of the 120 deaths that had data on gender and age (between 2013 and 2015), 57% occurred among males and 78% occurred among patients aged 5 years and above (Table 5).

**Table 5:** Characteristics of suspected rabies deaths by gender, age and region; 2013–2015.

PERIOD	NUMBER OF RABIES DEATHS BY REGION				OVERALL
	Central	Eastern	Northern	Western	
**2001–2015 (row %)**	135 (27.8)	139 (28.6)	130 (26.7)	82 (16.9)	**486 (100)**
**2013–2015 (col. %)**					
OVERALL	45 (100)	30 (100)	28 (100)	17 (100)	**120 (100)**
Male	24 (53.3)	18 (60.0)	17 (60.7)	9 (52.9)	**68 (56.7)**
Female	21 (46.7)	12 (40.0)	11 (39.3)	8 (47.1)	**52 (43.3)**
<5 years	11 (24.4)	6 (20.0)	6 (24.4)	3 (17.6)	**26 (21.6)**
≥5 years	34 (75.6)	24 (80.0)	22 (78.6)	14 (82.4)	**94 (78.3)**

**Fig 6:**
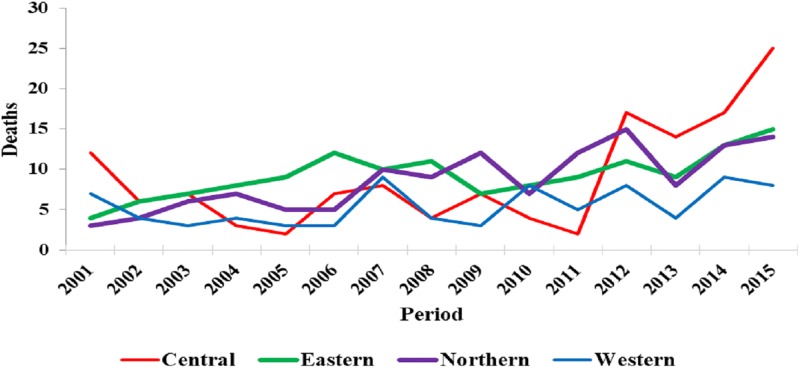
Trends of suspected human rabies deaths by region; 2001–2015.

From 2001 to 2015, there was a significant increase in cases of suspected rabies deaths in the Central (P = 0.02), Eastern (P = 0.002) and Northern regions (P<0.001) (Fig 6. Trends of suspected human rabies deaths by region; 2001–2015). The increase in suspected rabies deaths was not statistically significant in the Western region (P = 0.09).

## Discussion

Surveillance data collected between January 2001 and June 2015, showed an increase in trend of incidence of animal bite injuries in all regions of the country. The majority of patients with animal bite injuries were male patients and persons aged above 5 years. Districts in the Northern region had higher incidence of animal bites, compared to districts in other regions, with most affected districts located in the Northern region.

In absence of accurate data on trends of human rabies and challenges in rabies diagnosis in developing countries, use of surveillance data on animal bites provides useful information to support rabies surveillance and to improve allocation of medical and veterinary resources [7]. In Uganda where rabies is endemic, the increasing incidence of animal bite injuries suggests that potential exposure to rabies infection remains an important public health challenge. These results are similar to those reported in a previous cross-sectional study which showed that the burden of animal bites in Uganda is considerable [21]. Although some studies have documented increasing human and dog populations as possible explanations for the increasing incidence of animal bites [30], the role played by these factors in Uganda is unknown. Uganda conducted a livestock census in 2008 which estimated a dog population of 1.6 million dogs country wide [29] but to our knowledge, no follow-up survey has ever been conducted to update dog statistics or predict growth rates. However, countries in the same geographical region as Uganda have reported dog growth rates between 4.7% and 10% [31–33].

The finding that most of the animal bite injuries in our study were inflicted on males is consistent with results reported from other studies conducted elsewhere [34–36]. Although several factors may explain gender differences in the distribution of animal bites, occupational activities that expose individuals to dog bites are likely to be more common among males compared to females [36]. Although human activities associated with dog bites in Uganda have not been well studied, men often engage in activities that involve close interaction with dogs such as hunting and herding. Some studies have hypothesized that dog bites are more common among males because they are more adventurous and aggressive and females spend more time at home compared to males [37, 38].

The wide variation in the incidence of animal bite injuries in different districts and regions of the country may be due to several reasons. First and foremost, these regions had different reporting rates which may result in underreporting for districts with low reporting rates. Regional variations in background dog populations were documented by the Uganda national live stock census of 2008 and these may affect the distribution of animal bites [29]. A comparison of regional dog populations with the incidence of animal bites showed that the Eastern region, which had the lowest incidence of animal bites, had the lowest dog population. Similarly, the Northern region, which had the highest incidence of animal bites, had the highest dog population. Variations in the incidence of animal bites by region may also be explained by the geographical differences in these regions, population density and local epidemiology of rabies [39].

Previous findings from a study conducted in Uganda by Fevre et al found that 94% of animal bites were inflicted by dogs [21]; which were similar to results from another study in Chad where up to 97% of animal bites were inflicted by dogs [40]. Based on these findings in Uganda [21], we estimate that up to 196,000 animal bites were inflicted by dogs during the 15 year study period; and of these 150,920 bites by suspected rabid dogs. However, Fevre’s study might have overestimated the number of rabid dog bites since the probability of the biting dog was rabid was based on information provided by patients. If no post exposure vaccination is given to victims of rabid animal bites, the number of deaths that can result from rabid dog bites is huge. Fever et al used the Cleaveland et al model and estimated that in absence of post exposure vaccination, 6,602 bites from rabid dogs would result in 592 deaths [7, 21]. Using similar model assumptions, the estimated rabid animal bites reported in our study would result in 13,533 deaths, which is lower than the actual deaths reported in this study. Improved availability and access to rabies post-exposure prophylaxis over the last 10 years might have played a key role in reducing rabies cases. Currently 111 public hospitals and 179 level IV health centres are able to provide rabies post-exposure vaccination compared to the 10 hospitals in 2005 [21, 41]. However, it should be noted that the underreporting rate of rabies in African countries is estimated to be 160 times higher than what is officially reported in developed countries [42]. Rabies underreporting is mainly due to poor surveillance and difficulties in diagnosing rabies infection and it results in underestimation of the actual burden of the disease in these countries [6]. Therefore, the number of deaths captured through routine surveillance in Uganda may represent only a fraction of the true burden of the disease.

Analysis of the geographical distribution showed that while the incidence of animal bite injuries increased significantly in all the regions of Uganda, rabies deaths significantly increased in the Central, Eastern and Northern regions. This was likely to be due to regional differences in reporting of weekly data from district to national level. Between 2001 and 2011, Uganda was using a paper based surveillance system where hard copies of HMIS reports had to be delivered from districts to MoH headquarters. Some districts, particularly those that are farther from Ministry of Health headquarters experienced challenges in delivering their surveillance reports which impacted on reporting. From 2001 to 2011, the average reporting rate from districts in Western region was 65% compared to 74% in Eastern, 74% in Northern and 68% in Central region.

Eliminating rabies, amidst challenges of poor funding of health budgets and poor surveillance systems, is a difficult but a potentially achievable task in developing countries in Africa. For example, no single case of human rabies was recorded in South Africa in 2011 and in Tanzania in 2013, after years of implementation of rabies control programmes in these countries [23, 43]. Rabies elimination in Uganda is still difficult because current prevention and control efforts are centred around post exposure prophylaxis among humans. Vaccinations among domestic dogs and cats are mainly done voluntarily by a few owners or during mass vaccination campaigns in selected districts.

An effective strategy for combating rabies and dog bites in Uganda should focus on strengthening multi-sectoral collaboration and coordination through a “One Health” approach and strengthening rabies and animal bites surveillance as recommended during the 2015 global rabies conference [44]. Strong collaboration and coordination between human and animal sectors have been instrumental in successful control of human rabies in selected areas in Africa and Asia [43, 45]. Poor surveillance and lack of collaboration among partners can lead to poor understanding and underestimation of the burden of rabies [4, 6].

Improving animal bite and rabies surveillance should be one of the key strategies for rabies prevention and control. Animal bites remain the most important source of human rabies infection [6]. Therefore, rabies surveillance should be aligned to capture and report key epidemiological information such as actual age, residence and type of animal bite. This information is essential in guiding rabies prevention and control programs in settings where rabies is endemic. Laboratory diagnostic capacity for rabies should occupy a central role in supporting rabies surveillance and other efforts to combat rabies [46] yet this is still a challenge in many developing countries. Therefore, there is a need to establish rabies diagnostic capacity in the country; to be able to report on rabies confirmed cases. Improved rabies surveillance will ultimately lead to improvements in timely response, identification of areas where to focus rabies control interventions and monitoring the impact of control interventions [47].

This study had limitations. First and foremost, the data analyzed in this study was collected from a passive surveillance system and the results are subject to reporting biases such as underreporting arising from failure of districts to deliver HMIS reports to the MoH and some patients not reporting to health facilities that report to the national database. Therefore, there is a high likelihood that the animal bites and suspected rabies cases reported in this study are an underestimate of the actual burden. Secondly, age categories used for surveillance were very wide, making it difficult to assess age as a risk factor. Thirdly, the actual location (residence) of cases and data on the biting animal was not reported yet this information is important in strategic planning of effective responses and interventions. Lastly, the diagnosis of rabies in Uganda is mainly done on clinical grounds and as such, some of the reported deaths due to rabies may have been due to other similar diseases or conditions.

In conclusion, this study provides evidence that exposure to potential rabid animal bites and mortality attributed to rabies infection remain serious public health challenges across all regions of Uganda. There is need to strengthen and scale-up rabies prevention and control measures in Uganda. The control strategies should be built on the “One Health” approach with special focus on strengthening the rabies surveillance system to be able to collect more reliable data, controlling rabies in dogs and ensuring availability of post exposure prophylaxis at lower health facilities. The most affected areas, such as Northern Uganda, Kampala and Wakiso districts, should be prioritized during planning of prevention and control programmes.
